# Conditioned Medium from Mesenchymal Stem Cells Alleviates Endothelial Dysfunction of Vascular Grafts Submitted to Ischemia/Reperfusion Injury in 15-Month-Old Rats

**DOI:** 10.3390/cells10051231

**Published:** 2021-05-17

**Authors:** Sevil Korkmaz-Icöz, Xiaoxin Sun, Shiliang Li, Paige Brlecic, Sivakkanan Loganathan, Mihály Ruppert, Alex Ali Sayour, Tamás Radovits, Matthias Karck, Gábor Szabó

**Affiliations:** 1 Department of Cardiac Surgery, University Hospital Heidelberg, 69120 Heidelberg, Germany; xiaoxin_sun1990@163.com (X.S.); lishiliangcom@hotmail.com (S.L.); paigebrlecic@yahoo.com (P.B.); sivakkanan@gmail.com (S.L.); ruppertmis@gmail.com (M.R.); alexali.sayour@gmail.com (A.A.S.); matthias.karck@med.uni-heidelberg.de (M.K.); Gabor.Szabo@uk-halle.de (G.S.); 2 Department of Cardiac Surgery, University Hospital Halle (Saale), 06120 Halle, Germany; 3 Heart and Vascular Center, Semmelweis University, 1122 Budapest, Hungary; radovitstamas@yahoo.com

**Keywords:** ischemia/reperfusion, endothelial function, mesenchymal stem cells, conditioned medium, caspase-12

## Abstract

In patients undergoing coronary artery bypass grafting (CABG), ischemia/reperfusion injury (IRI) is the main contributor to organ dysfunction. Aging-induced vascular damage may be further aggravated during CABG. Favorable effects of conditioned medium (CM) from mesenchymal stem cells (MSCs) have been suggested against IRI. We hypothesized that adding CM to saline protects vascular grafts from IRI in rats. We found that CM contains 28 factors involved in apoptosis, inflammation, and oxidative stress. Thoracic aortic rings from 15-month-old rats were explanted and immediately mounted in organ bath chambers (aged group) or underwent 24 h of cold ischemic preservation in saline-supplemented either with vehicle (aged-IR group) or CM (aged-IR+CM group), prior to mounting. Three-month-old rats were used as referent young animals. Aging was associated with an increase in intima-to-media thickness, an increase in collagen content, higher caspase-12 mRNA levels, and immunoreactivity compared to young rats. Impaired endothelium-dependent vasorelaxation to acetylcholine in the aged-IR group compared to the aged-aorta was improved by CM (aged 61 ± 2% vs. aged-IR 38 ± 2% vs. aged-IR+CM 50 ± 3%, *p* < 0.05). In the aged-IR group, the already high mRNA levels of caspase-12 were decreased by CM. CM alleviates endothelial dysfunction following IRI in 15-month-old rats. The protective effect may be related to the inhibition of caspase-12 expression.

## 1. Introduction

In patients undergoing coronary artery bypass grafting (CABG), ischemia/reperfusion injury (IRI) is the main contributor to organ dysfunction or failure. Hypothermia and hypoxic insult induce vascular graft injury; however, reperfusion itself may paradoxically augment tissue damage originally produced by ischemia alone [1]. The mechanisms of cold ischemia/warm reperfusion-elicited cell/tissue injury are combinations of both hypoxia/reoxygenation and hypothermia/rewarming injuries. During reperfusion, polymorphonuclear leucocytes accumulate in the ischemic tissue, reactive oxygen species (ROS) and reactive nitrogen species (RNS) are generated [2]. ROS generation can disrupt endoplasmic reticulum (ER) function, consequently leading to vascular dysfunction, altered tissue barrier functions, and even apoptosis [3].

It is well-established that normal aging is associated with changes in vascular function and structure [4]. We have previously reported that aging induces alterations in endothelium-dependent relaxation and vasocontractile responses [5,6]. Thus, pre-existent vascular damage due to aging may be aggravated by hypothermic preservation/reperfusion during CABG, and these patients may run a high risk for cardiovascular complications. As a consequence of the increasing number of older coronary artery disease patients, optimized intra-operative storage of the vascular graft prior to and during CABG is of particular interest for surgeons. Currently, storage of bypass grafts with physiological saline solution is the clinical routine [7]; however, preventing the adverse effects of IRI during the CABG procedure remains a problem.

Bone marrow-derived mesenchymal stem cells (MSCs), multipotent cells, showed significant immunomodulatory, anti-inflammatory, and tissue repair properties in preclinical and ongoing clinical trials [8]. Initially, MSCs’ ability to differentiate into cardiomyocytes, endothelial cells, and vascular smooth muscle cells, or their cell-to-cell contacts was proposed as the principal mechanism underlying their therapeutic effects. Even more promising, as the magnitude of newly differentiated cells after MSCs transplantation is too low to explain such effects, paracrine factors released by MSCs, including a combination of growth factors, chemokines, and cytokines [9], have proven to be important mediators of cardioprotection [10,11]. Accordingly, treatment with conditioned medium (CM) from bone marrow-derived MSCs was found to reduce myocardial apoptosis and oxidative stress following IRI [12]. We have demonstrated that the perfusion of 15-month-old donor rat hearts with CM protects against myocardial IRI in a model of heterotopic heart transplantation [13]. Our recent study shows that the preservation of aortic rings from brain-dead rats with CM protects vascular grafts against in vitro IRI [14]. Therefore, the factors secreted by MSCs could be used to extend the list of therapeutic targets for vascular graft protection against IRI.

Taken together, in the present study, we hypothesized that adding CM to physiological saline solution protects vascular grafts from IRI in 15-month-old rats. Furthermore, because ER stress exacerbates IRI-induced apoptosis, we investigated CM’s link to caspase-12 expression, a representative molecule related to the ER stress-induced cell death signaling pathway.

## 2. Materials and Methods

See the Supplementary Materials for further details.

### 2.1. Animals

Male Lewis rats, obtained from Janvier Labs (Saint Berthevin, France), were housed in controlled rooms (22 ± 2 °C with 12–12 h light-dark cycles) and acclimatized for 1 week. They were fed a standard laboratory diet and given water ad libitum. All animals received humane care in compliance with the Principles of Laboratory Animal Care, formulated by the National Society for Medical Research, and with the Guide for the Care and Use of Laboratory Animals, prepared by the Institute of Laboratory Animal Resources and published by the National Institutes of Health (NIH Publication, 8th Edition, 2011) [15] with prior approval (on 06 October 2014) by theregional authorities in Karlsruhe, Germany (G183/14).

### 2.2. Preparation of Bone Marrow-Derived MSCs-CM

CM was prepared from young rats (8–12 week-old), as previously reported [1,6]. Briefly, both femurs and tibias were harvested, and bone marrow MSCs were isolated by flushing with Dulbecco’s phosphate-buffered saline (DPBS) (Sigma, St. Louis, MO, USA). The MSCs were suspended in MSC Expansion Medium (R&D System, Minneapolis, MN, USA) and then incubated at 37 °C with 5% CO2 on cell culture flasks. When cultures have reached about 80% confluency, primary cells were subcultured 1:3. When Passage 3, MSCs reached greater than 80% confluency, the medium was aspirated, and MSCs were washed 3 times with DPBS. Then, Dulbecco’s modified Eagle’s medium (D-MEM) (Life Technologies, Grand Island, NY, USA) was added to culture dishes containing MSCs and placed into the incubator for 24 h. Primary CM was collected and further concentrated at 4500× *g* for 4 h at 4 °C by ultrafiltration. The protein concentration of the CM was measured by Bradford protein assay and used at a final concentration of 0.5 mg/mL. D-MEM was used as a control (nonconditioned medium).

### 2.3. Antibody Array

For the simultaneous detection of the relative expression of 90 target proteins in CM, RayBio^®^ Biotin Label-based rat antibody array 1 (BioCat GmbH, Heidelberg, Germany) was used according to the manufacturer’s guidance and instructions.

### 2.4. Rat Model of Endothelial Dysfunction Induced by Cold Ischemic Storage and Reperfusion

The protocol has previously been described in detail [16,17,18].

#### 2.4.1. Preparation and Conservation of Aortic Rings

The rats were anesthetized with sodium pentobarbital intraperitoneally at a dose of 60 mg/kg and placed on controlled heating pads, maintaining their core temperature (measured via a rectal probe) at 37 °C. The descending thoracic aorta was carefully explanted and quickly transferred to cold (+4 °C) Krebs-Henseleit-solution (KHL) containing 118 mM NaCl, 4.7 mM KCl, 1.2 mM KH_2_PO_4_, 1.2 mM MgSO_4_, 1.77 mM CaCl_2_, 25 mM NaHCO_3_, and 11.4 mM glucose (pH = 7.4). The aorta was isolated, cleaned of periadventitial fat and surrounding connective tissue under a microscope, and it was cut into 4-mm wide rings.

#### 2.4.2. Experimental Groups

The thoracic aortic rings were stored for 24 h at 4 °C in closed, air-free tubes filled with physiological saline-supplemented with either vehicle (young-IR (*n* = 28 rings, 7 rats) and aged-IR (*n* = 39 rings, 10 rats) groups) or CM (young-IR+CM (*n* = 30 rings, 8 rats) and aged-IR+CM (*n* = 31 rings, 8 rats) groups). The tubes were previously equilibrated with nitrogen, extruding oxygen from the solution. After 24 h of cold ischemic conservation, the rings proceeded to the organ bath. To simulate free radical burst and endothelial injury, which usually occurs during reperfusion in vivo, 200 µM of hypochlorite were added to the baths for 30 min. Aortic rings in the young (*n* = 27 rings, 7 rats) and aged (*n* = 28 rings, 8 rats) normoxia-groups did not undergo cold ischemic storage but were immediately mounted in organ baths.

#### 2.4.3. Ex Vivo Organ Bath Experiments

The aortic rings were mounted on a stainless steel hook and subjected to a passive tension of 2 g in organ baths (Radnoti Glass Technology, Monrovia, CA, USA), containing 30 mL of KHL and continuously gassed with 95% O_2_-5% CO_2_ at 37 °C. The tissue was equilibrated for 60 min with a change of KHL every 30 min as a precaution against interfering metabolites. During this period, the tension was periodically adjusted to 2 g. At the beginning of each experiment, potassium chloride (KCl, 80 mM) was used to test the viability and to prepare the vessel rings for stable contractions and reproducible dose-response curves to other vasoactive agents. This was maintained for approximately 30 min, after which the aortic rings were washed until resting tension was again obtained. An α-adrenergic receptor agonist, phenylephrine (PE, 10^−9^–10^−5^ M), was used to precontract the rings until a stable plateau was reached, and the relaxation responses were examined by adding cumulative concentrations of the endothelium-dependent vasorelaxant acetylcholine (ACh, 10^−9^–10^−5^ M) and endothelium-independent dilator sodium nitroprusside (SNP, 10^−10^–10^−5^ M). The relaxation is expressed as the percentage of contraction induced by PE.

### 2.5. Aortic Histomorphometry

Aortic segments were fixed with 4% buffered paraformaldehyde solution and embedded in paraffin. Then, 5-µm thick sections were placed on adhesive slides and stained with hematoxylin and eosin as described elsewhere [19].

### 2.6. Acid Fuchsin Orange (AFOG) Staining

AFOG staining was used to detect collagen fibers in the aortic tissue as described elsewhere [20]. The collagen content was determined by semi-quantitative morphometry scoring of the sections under a microscope using Cell^A software (Olympus Soft Imaging Solutions GmbH, Munster, Germany).

### 2.7. Quantitative Real-Time Reverse Transcription Polymerase Chain Reaction (PCR) Analysis

Caspase-12 is located in the ER and is responsible for ER stress-induced apoptosis. As previously reported [20], total RNA was isolated with the RNeasy Fibrous Tissue Mini Kit (Qiagen, Hilden, Germany) from frozen distal regions of the aortic tissue according to manufacturer instructions.

### 2.8. Caspase-12 Immunolabeling

Immunoreactivity to caspase-12 (1:100; Novus Biologicals, Littleton, CO, USA) was tested on buffered paraformaldehyde solution (4%) fixed, paraffin-embedded 5-µm thick aortic sections.

### 2.9. Tibial Lengths

Tibial lengths were measured using micrometer calipers.

### 2.10. Statistical Analysis

All data are expressed as the mean ± standard error of the mean (SEM). Statistical analyses were performed using GraphPad Prism 7.02 software (GraphPad Software, Inc., San Diego, CA, USA). The Shapiro–Wilk normality test was used to assess deviations from normal distribution before statistical tests were applied. For data with normal distribution, a two-sample Student’s *t*-test was used to analyze the differences between the young and aged groups. In case of non-normal distribution, a nonparametric Mann–Whitney U test was applied. In all other cases, one-way ANOVA followed by Tukey’s post hoc test was carried out for multiple comparisons. If the data were non-normal, the nonparametric Kruskal–Wallis test followed by Dunn’s post hoc test was used to investigate intergroup differences. A value of two-tailed *p* < 0.05 was considered statistically significant.

## 3. Results

### 3.1. Characterization of 15-Month-Old Rat Model

#### 3.1.1. Body Weight and Aortic Morphometry

**Figure 1:**
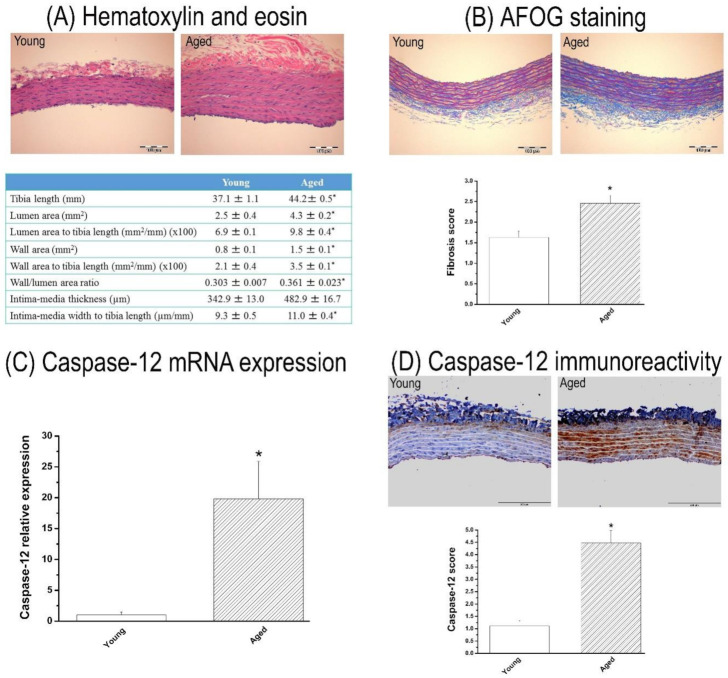
Characterization of the thoracic aorta in 15-month-old rats. Representative image of aortic cross-section stained with (**A**) hematoxylin and eosin followed by the aortic morphometric measurements and (**B**) acid fuchsin orange G (AFOG) staining (×20, bar = 100 µm) followed by semi-quantitative analysis of aortic fibrosis. Caspase-12 (**C**) mRNA expression and (**D**) immunoreactivity (×20, bar = 200 µm) in the aorta. Data are represented as mean ± SEM. * *p* < 0.05 vs. young. *n* = 7–8 rats/group.

Aged rats showed significantly higher body weight compared with the control group (560 ± 2 vs. 364 ± 8 g, *p* < 0.001). Morphometrical analyses of the aortas revealed that wall thickness, wall cross-section area, the lumen area normalized to tibial length, and the wall:lumen area ratio were significantly higher in the aged compared to the young group (Figure 1A).

#### 3.1.2. Fibrosis in the Aorta

In the aged group, the histological fibrosis score, assessed in AFOG-stained sections, was significantly higher than in the young group (Figure 1B).

#### 3.1.3. Caspase-12 Expression in the Aorta

Both mRNA levels and immunoreactivity of caspase-12 were significantly increased in the aortic wall of aged rats when compared to the young group (Figure 1C,D).

#### 3.1.4. Contractile and Relaxant Responses in the Aortic Rings

**Figure 2:**
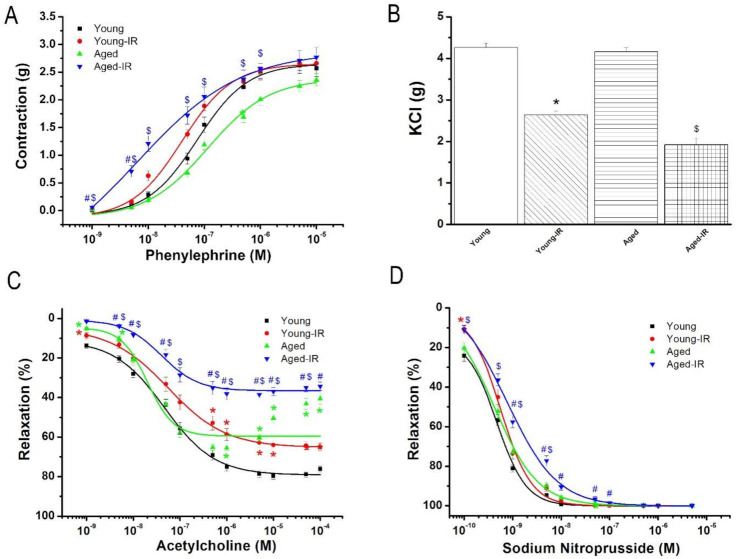
Effect of ischemia/reperfusion (IR) injury on contractile and relaxation responses. Contractile responses (**A**) for phenylephrine and (**B**) to high potassium K^+^-induced depolarization, and (**C**) acetylcholine-induced endothelium-dependent and (**D**) for sodium nitroprusside-induced endothelium-independent vasorelaxation of isolated thoracic aortic rings in young and 15-month-old rats. KCl indicates potassium chloride. Data are represented as mean ± SEM. * *p* < 0.05 vs. young, ^#^ *p* < 0.05 vs. young-IR, ^$^ *p* < 0.05 vs. aged.

Whereas aging did not alter maximal contractile responses to PE, to high K^+^-induced depolarization, and maximal relaxation responses to endothelium-independent relaxation with SNP, it did significantly decreased endothelium-dependent vasorelaxation to ACh compared to the young group (Figure 2).

### 3.2. Effect of IRI in Young and Aged Rats’ Aorta

#### 3.2.1. Effect of IRI on Contractile Responses of Aortic Rings

Exposure of aortic segments to PE (10^−9^ M–5 × 10^−5^ M) led to a concentration-dependent increase in tension (Figure 2A). While IRI had no effect on contractile responses to PE among the experimental groups, it significantly decreased the vasoconstrictive responses to KCl in both young and aged groups compared to their respective controls (Table 1, Figure 2A,B). The aortas’ sensitivity (pD_2_-value) to PE was significantly greater in the young-IR and aged-IR groups compared with their corresponding controls (Table 1).

#### 3.2.2. Effect of IRI on Endothelium-Dependent Vasorelaxation of Aortic Rings

In aortic rings precontracted with 10^−6^ M PE, 10^−9^ M–10^−4^ M ACh-induced concentration-dependent relaxation in all experimental groups (Figure 2C). IRI significantly decreased ACh-induced relaxation in both young and aged groups compared with their respective controls. The adverse impact of IRI on R_max_ to ACh was significantly increased in aged aortic rings compared to young ones, according to normalization to youngs (difference of R_max_ to ACh ratio: aged-IR/aged 41.2 ± 3.5% vs. young-IR/young 18.6 ± 2.4%, *p* < 0.001). Endothelium-dependent vasorelaxation to ACh was further impaired in the aged-IR group compared to the young-IR group (Figure 2C). IRI had no effect on the aortic rings’ sensitivity to ACh (Table 1).

#### 3.2.3. Effect of IRI on Endothelium-Independent Vasorelaxation of Aortic Rings

Figure 2D shows concentration-dependent relaxation induced by 10^−10^ M–10^−5^ M SNP, an endothelium-independent vasodilator. The concentration-response curve to SNP in aortas from the aged-IR group was shifted to the right compared with the aged group; however, no difference in the maximal endothelium-independent relaxation was observed (Figure 2D, Table 1).

#### 3.2.4. Effect of IRI on Caspase-12 mRNA Expression and Immunoreactivity in the Aorta

**Figure 3:**
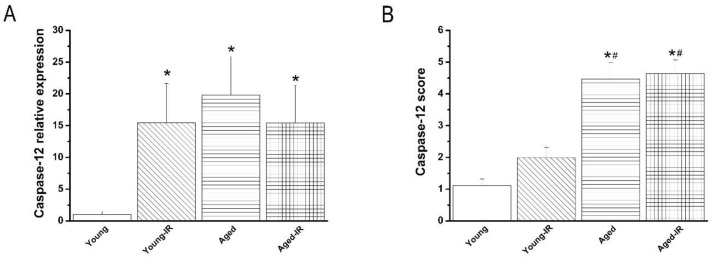
Effect of ischemia/reperfusion (IR) injury on caspase-12 expression. Caspase-12 (**A**) mRNA expression and (**B**) immunoreactivity in the aorta in young and 15-month-old rats. Data are represented as mean ± SEM. * *p* < 0.05 vs. young, ^#^ *p* < 0.05 vs. young-IR.

Caspase-12 mRNA expression was significantly increased (Figure 3A), and the immunoreactivity had a tendency to be higher (without reaching statistical significance) (Figure 3B) in the young-IR compared to the young group. However, IRI has no further effect on already high levels of caspase-12 in the aged group (Figure 3A,B).

### 3.3. Effect of CM Against IRI in Young and Aged Rats’ Aorta

#### 3.3.1. Characterization of the CM

As we already reported [13], antibody array against 90 specified rat proteins identified 28 factors in CM that are involved in apoptosis, inflammation, or oxidative stress, including tissue inhibitor of metalloproteinase-1, growth hormone/growth hormone receptor, endocrine gland-derived vascular endothelial growth factor, vascular endothelial growth factor, activin A, tumor necrosis factor-related apoptosis-inducing ligand, thrombospondin, TROY, metalloproteinase-1, fibroblast growth factor-binding protein, neuropilin-2, platelet-derived growth factor-AA, monocyte chemoattractant protein-1, interferon gamma-induced protein, cytokine-induced neutrophil chemoattractant 2/3, fibroblast-stimulating lipopeptide-1, macrophage-derived chemokine, macrophage migration inhibitory factor, macrophage inflammatory protein-1, osteopontin/secreted phosphoprotein 1, osteopontin, granulocyte-macrophage colony-stimulating factor, interferon gamma-induced protein, lipopolysaccharide-induced chemokine, insulin-degrading enzyme, tissue inhibitor of metalloproteinase-3, and metalloproteinase-13.

#### 3.3.2. Effects of CM on Contractile Responses After IRI

**Figure 4:**
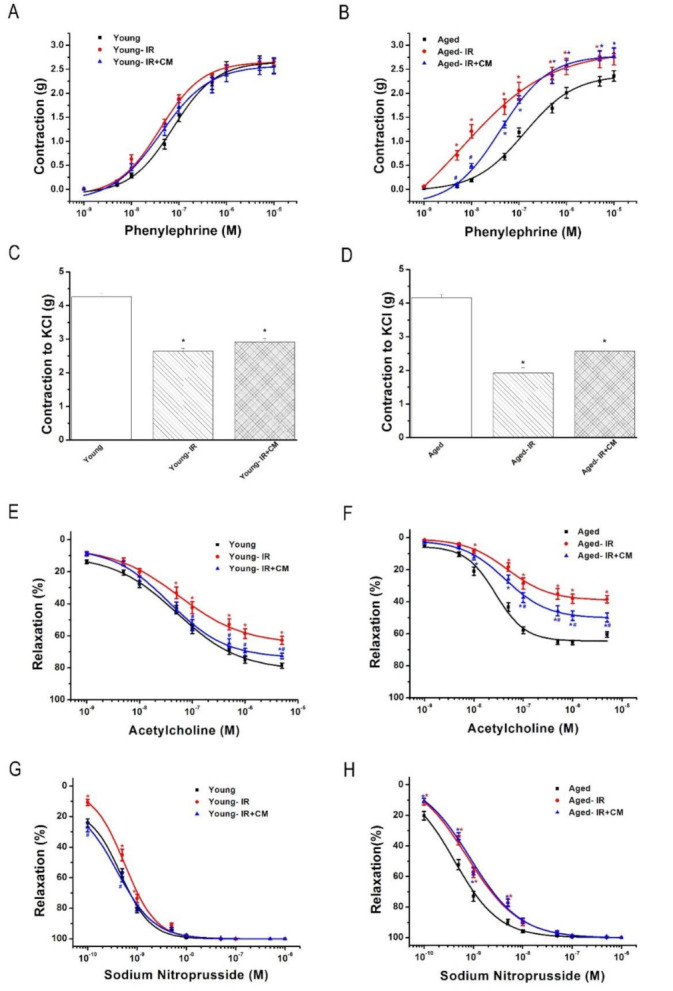
Effects of conditioned medium (CM) from bone marrow-derived mesenchymal stem cells against ischemia/reperfusion (IR) injury on contractile and relaxation responses in young and in 15-month-old rats. Contractile responses (**A**,**B**) for phenylephrine and (**C**,**D**) to high potassium K^+^-induced depolarization, and (**E**,**F**) acetylcholine-induced endothelium-dependent and (**G**,**H**) sodium nitroprusside-induced endothelium-independent vasorelaxation of isolated thoracic aortic rings from young and 15-month-old rats. KCl indicates potassium chloride. Data are represented as mean ± SEM. * *p* < 0.05 vs. corresponding young or aged, ^#^ *p* < 0.05 vs. corresponding young-IR or aged-IR.

The preservation of aortic rings with CM had no effect on the vasoconstrictive response to PE, nor on IR-induced decreased maximal contractile response to high K^+^-induced depolarization in both young and aged groups (Table 2, Figure 4A–D).

#### 3.3.3. Effects of CM on Endothelium-Dependent Vasorelaxation After IRI

The preservation of aortic rings with CM significantly improved IR-induced decreased endothelium-dependent vasorelaxation in response to ACh in both young and aged groups (Table 2, Figure 4E,F).

#### 3.3.4. Effects of CM on Endothelium-Independent Vasorelaxation After IRI

There was no significant difference in the maximal endothelium-independent vasorelaxation to SNP among the three groups in both young and aged rats. The preservation of aortic rings with CM had no effect on IR-induced shift to the right of the concentration-response curves for SNP in the aged group. However, CM significantly increased the sensitivity of pD_2_ to SNP in the young group (Table 2, Figure 4G,H).

#### 3.3.5. Effects of CM on Caspase-12 Expression After IRI

**Figure 5:**
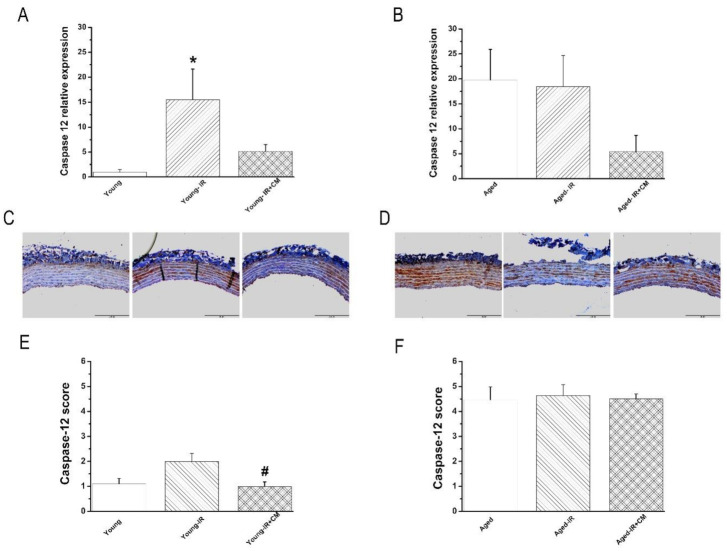
Effects of conditioned medium (CM) from bone marrow-derived mesenchymal stem cells against ischemia/reperfusion (IR) injury on caspase-12 expression. (**A**,**B**) caspase-12 mRNA expression and (**C**,**D**) representative images of caspase-12 immunohistochemical staining (brown staining, ×20, bar = 200 µm) followed by (**E**,**F**) semi-quantitative analysis in young and 15-month-old rats. Data are represented as mean ± SEM. * *p* < 0.05 vs. young, ^#^ *p* < 0.05 vs. young-IR.

CM-preserved young-IR aortic rings showed similar mRNA levels of caspase-12 when compared with the young group (Figure 5A). Furthermore, caspase-12 immunoreactivity was significantly decreased in the young-IR+CM compared to the young-IR rats (Figure 5C–E). Already high caspase-12 mRNA expression in the aged-IR group had a tendency to be lower in the aged-IR+CM aortas (Figure 5B). However, already elevated levels of caspase-12 immunoreactivity did not decrease after treatment with CM in the aged-IR groups (Figure 5D–F).

## 4. Discussion

In the present study, we tested the hypothesis that adding CM to physiological saline protects vascular grafts from IRI in 15-month-old rats. We have shown that in vitro preservation of both young and aged vascular grafts with CM alleviates endothelial dysfunction after IRI in rats. The protective effect may be related to the inhibition of caspase-12.

Vascular grafts are essential therapeutic materials for bypass surgery. The endothelium has a key role in the regulation of vascular homeostasis through the release of endothelium-derived relaxing factors, such as nitric oxide (NO), prostacyclin (PGI_2_), and endothelium-derived hyperpolarizing factor (EDHF), as well as vasoconstrictive factors, such as endothelin and thromboxane. This can be important for the control of vascular tone. Without adequate protection, both aging-related structural and functional changes and prolonged hypothermic storage of blood vessels would contribute to further endothelial dysfunction and endothelial cell activation. This can lead to leukocyte adhesion and migration into the vessel wall, platelet aggregation, vascular smooth muscle cell proliferation, increased vasoconstriction [21], and subsequently to reduced early patency of implanted vascular grafts. Endothelial dysfunction can be defined by impaired vasodilation to stimuli, such as shear stress or ACh, and by proinflammatory and prothrombic status. In the present study, we have shown that IRI impaired endothelium-dependent vasorelaxation to a substantially greater extent in aortic rings of 15-month-old rats, as compared with those from younger ones. This might indicate that aging is associated with reduced ischemic tolerance of vascular grafts. Therefore, new therapeutic strategies are required to protect the endothelium of vascular grafts in conditions of increased oxidative stress, such as aging and prolonged ischemia/reperfusion, to preserve vascular function and to improve the long-term survival after CABG. To improve ex vivo graft preservation, different antioxidant treatments have been investigated during organ preservation to reduce the injury [4]. Furthermore, the incorporation of antioxidant compounds within implanted biomaterials should be able to regulate the oxidative stress level and protect tissue recovery [5]. In the current clinical settings, there remains a great need for better organ preservation solutions, which can protect organs against IRI, minimizing its adverse effects on vascular graft function. In the present study, we therefore investigated the effects of physiological saline enriched with CM in an in vitro experimental model of vascular dysfunction induced by IRI. The endothelial function was evaluated by applying ACh on PE-precontracted aortic rings. We demonstrated that exposure of aortic rings to prolonged cold ischemia/warm reperfusion resulted in endothelial dysfunction in both young and aged rats, which can be prevented by CM. CM-derived from MSCs was shown to secrete a large number of chemokines, cytokines, growth factors, and other substances that promote protection. In the current study, we identified that CM contains tissue inhibitors of metalloproteinase (TIMP)-1, growth hormone, prokineticin, vascular endothelial growth factor (VEGF), and activin A factors, which may confer protection and contribute to an improved functional outcome after vascular IRI. TIMP-1, an inhibitor of MMPs, has been shown to display anti-apoptotic properties and indirectly induce cell signaling [22]. Additionally, it has been demonstrated that growth hormone deficiency in rats leads to reduced functional activity of the muscarinic receptors in response to ACh in the coronary vascular bed [23]. Furthermore, it is known that VEGF can serve as an in vitro survival factor for vascular endothelium [24,25]. Activin A is a transforming growth factor (TGF)-ß superfamily glycoprotein hormone, and the members of this superfamily are involved in different cellular processes, such as apoptosis [26]. It has been reported that the incubation of hypoxic primary human aortic endothelial cells with CM from multipotent stromal cells inhibited apoptosis and increased cell survival. The secretion of angiogenic and anti-apoptotic factors, such as IL-6, VEGF, and MCP-1, by MSCs partly contributed to the observed protective effect [27]. In accordance with these results, our data suggest that the “cocktail” of MSCs-derived soluble factors identified in CM may contribute to tissue preservation, thereby improving vascular graft function after IRI. Further experimental research is required to elucidate the role of other factors identified in CM from MSCs against vascular IRI injury in 15-month-old rats.

### 4.1. Mechanisms Underlying the Protective Effects of CM after IRI in Vascular

Apoptosis-mediated cell death is a key factor involved in the pathogenesis of IRI, leading to tissue damage. There is increasing evidence that ER stress plays a crucial role in IR-induced cell dysfunction [28], and various stimuli, such as oxidative stress, can lead to endothelial dysfunction, in part through the activation of ER stress [29]. The cellular response to ER stress is known as the unfolded protein response, a protective and well-established signaling cascade that is activated by the ER’s early stress response. However, when an attempt to overcome ER stress fails, i.e., the unfolded protein response is not enough to promote survival, the ER induces apoptosis by promoting the expression of apoptosis-inducing factors, such as members of the caspase family. Because ER stress increases caspase-12 levels, we sought to evaluate the protective action of CM against aging or IRI-induced caspase-12 expression. We found that both caspase-12 mRNA expression and immunoreactivity were reduced in the young-IR group by CM. However, in the aged group, even though CM decreased mRNA levels of caspase-12, it had no effect on already high level of caspase-12 immunoreactivity. This demonstrated that the attenuation of caspase-12 expression, in part, protects against endothelial dysfunction, however other important pathways seem to be involved in the beneficial effect CM has on vascular grafts of aged rats. It has been shown that bone marrow stromal cell therapy inhibits the expression of caspase-12 in rats with ischemic spinal cord injury [30]. It has also been demonstrated that activin A inhibited ER stress-induced apoptotic and autophagic cell death [31]. Even though this study does not provide direct mechanistic evidence that factors characterized from CM, such as activin A, VEGF, TIMP-1, growth hormone, and prokineticin, are in fact responsible for the improved endothelial function, it does show that caspase 12 expression was down-regulated.

### 4.2. Study Limitations

First, the thoracic aorta was studied using an ex vivo vascular ring apparatus to examine vascular reactivity. The involvement of non-aortic tissues, blood flow to the tissues, and activation of leukocytes need to be translated into a clinically relevant in vivo situation. Even though the therapeutic approach of targeting caspase-12 was suggested to be partly responsible for the CM’s protective effect on endothelial dysfunction, other proteins involved in ER stress-induced cell death were not investigated. It remains unclear whether other essential pathways may also take part in this effect.

## 5. Conclusions

This study provides experimental evidence that the preservation of vascular grafts with CM alleviates endothelial dysfunction after IRI in both young and 15-month-old rats. This protective effect may be related to the inhibition of caspase-12. From the clinical point of view, in patients undergoing bypass surgery, IRI is the main contributor to organ dysfunction or failure. Aging-induced vascular damage may be further aggravated during bypass surgery and may lead to low patency rates. In current clinical settings, a great need for better organ preservation solutions against IRI remains, which can minimize IRI’s adverse effects on graft dysfunction. Various factors present in CM from bone marrow-derived MSC may represent a balanced “cocktail”, acting together to promote protection against endothelial dysfunction during bypass surgery and may be a novel approach in cardiovascular surgery.

## Figures and Tables

**Table 1:** Quantitative analysis of aortic vascular function after ischemia/reperfusion (IR) injury in young and aged rats.

	Young	Young-IR	Aged	Aged-IR
PE (g)	2.63 ± 0.15	2.66 ± 0.07	2.36 ± 0.11	2.77 ± 0.18
pD_2_ to PE	7.07 ± 0.04	7.45 ± 0.06 *	6.57 ± 0.22	8.35 ± 0.21 ^$^
KCl (g)	4.26 ± 0.11	2.64 ± 0.09 *	4.16 ± 0.10	1.92 ± 0.16 ^$^
R_max_ to ACh (%)	79.81 ± 1.41	65.01 ± 1.93 *	65.45 ± 1.40 *	38.49 ± 2.32 ^#,$^
pD_2_ to ACh	7.25 ± 0.08	7.05 ± 0.11	6.95 ± 0.27	6.81 ± 0.18
R_max_ to SNP (%)	100.0 ± 0.0	100.0 ± 0.0	100.0 ± 0.0	100.0 ± 0.0
pD_2_ to SNP	9.30 ± 0.02	9.26 ± 0.05	9.31 ± 0.06	9.14 ± 0.09 ^$^

Data are represented as mean ± SEM. PE indicates phenylephrine; KCl, potassium chloride; ACh, acetylcholine; SNP, sodium nitroprusside; R_max_, maximum relation, and pD_2_, negative logarithm of the corresponding half-maximum response (EC_50_). * *p* < 0.05 versus young; ^#^ *p* < 0.05 versus young-IR; ^$^ *p* < 0.05 versus aged.

**Table 2:** Quantitative analysis of aortic vascular function after treatment with conditioned medium (CM) from bone marrow-derived mesenchymal stem cells against ischemia/reperfusion (IR) injury in young and aged aortic rings.

	Young	Young-IR	Young-IR + CM
PE (g)	2.63 ± 0.15	2.66 ± 0.07	2.57 ± 0.17
pD_2_ to PE	7.07 ± 0.04	7.45 ± 0.06 *	7.30 ± 0.05 *
KCl (g)	4.26 ± 0.11	2.64 ± 0.09 *	2.92 ± 0.10 *
R_max_ to ACh (%)	79.81 ± 1.41	65.01 ± 1.93 *	72.63 ± 1.78 *^,#^
pD_2_ to ACh	7.25 ± 0.08	7.05 ± 0.11	7.23 ± 0.12
R_max_ to SNP (at 5 × 10^−7^ M) (%)	100.0 ± 0.0	100.0 ± 0.0	100.0 ± 0.0
pD_2_ to SNP	9.30 ± 0.02	9.26 ± 0.05	9.55 ± 0.10 ^#^
	**Aged**	**Aged-IR**	**Aged-IR + CM**
PE (g)	2.36 ± 0.11	2.77 ± 0.18 *	2.84 ± 0.10 *
pD_2_ to PE	6.57 ± 0.22	8.35 ± 0.21 *	7.34 ± 0.04 *
KCl (g)	4.16 ± 0.10	1.92 ± 0.16 *	2.57 ± 0.10 *
R_max_ to ACh (%)	65.45 ± 1.40	38.49 ± 2.32 *	53.89 ± 2.46 *^,#^
pD_2_ to ACh	6.95 ± 0.27	6.81 ± 0.18 *	7.03 ± 0.19 *
R_max_ to SNP (at 5 × 10^−7^ M) (%)	99.7 ± 0.2	99.7 ± 0.2	99.7 ± 0.2
pD_2_ to SNP	9.31 ± 0.06	9.14 ± 0.09 *	9.09 ± 0.07 *

Data are represented as mean ± SEM. PE indicates phenylephrine; KCl, potassium chloride; ACh, acetylcholine; SNP, sodium nitroprusside; R_max_, maximum relation, and pD_2_, negative logarithm of the corresponding half-maximum response (EC_50_). Data are represented as mean SEM. * *p* < 0.05 versus young or aged; ^#^ *p* < 0.05 versus young-IR or aged-IR.

## Data Availability

The data presented in this study are available on reasonable request from the corresponding author.

## Supplementary Materials

The following are available online at https://www.mdpi.com/article/10.3390/cells10051231/s1, Supplementary Materials and Methods: (acid fuchsin-orange staining, Quantitative real-time reverse transcription polymerase chain reaction (PCR) analysis, caspase-12 immunolabeling).
