# Supplementary material for: Conditioned Medium from Mesenchymal Stem Cells Alleviates Endothelial Dysfunction of Vascular Grafts Submitted to Ischemia/Reperfusion Injury in 15-Month-Old Rats
Source: Cells. 2021 May 17;10(5):1231. doi: 10.3390/cells10051231 (PMC8155879; doi:10.3390/cells10051231)
Supplement: Supplementary file 1 [file cells-10-01231-s001.zip › cells-1208548-supplementary.pdf]

MATERIALS AND METHODS

Acid fuchsin-orange (AFOG) staining

AFOG staining was used to detect collagen fibres in the aortic tissue as described elsewhere.[1] First, paraffin-embedded sections were deparaffinised by immersing them in xylene, and then rehydrated through 100% alcohol, 95% alcohol, then 70% alcohol. A brief washing in water followed. Samples were then refixed in Bouin's solution, stained in Weigert's iron hematoxylin working solution, and then differentiated several times in 1% HCl alcohol. The sections were washed in running tap water between each of the previously described steps. Then, the slides were treated with aqueous 1% phosphomolybdic acid solution, rinsed with distilled water, placed in AFOG staining solution, and washed in water. Each slide was subsequently dehydrated through graded alcohols. Aortic sections were finally soaked in xylene, and coverslips were applied using Permount. The collagen content was determined by semi-quantitative morphometry scoring of the sections under a microscope using Cell^A software (Olympus Soft Imaging Solutions GmbH) according to the following: 0: absent, 1: slight, 2: moderate, 3: intense. The evaluation was conducted by an analyst blinded to the experimental groups.

Quantitative real-time reverse transcription polymerase chain reaction (PCR) analysis

Caspase-12 is located in the ER and is responsible for ER stress-induced apoptosis. As previously reported[1], total RNA was isolated from frozen distal regions of the aortic tissue, using the RNeasy Fibrous Tissue Mini Kit (Qiagen, Hilden, Germany), after homogenisation according to manufacturer instructions. RNA concentration and purity were determined photometrically by measuring the absorbance at 260 nm, 282 nm, and 230 nm. RNAs were reverse transcribed into cDNA with Quantiscript Reverse Transcriptase (Quantitect Reverse Transcription Kit, Qiagen, Hilden, Germany) using 500 µg of RNA in a volume of 20 µl. Real-time PCR was performed on the LightCycler 480 system using the LightCycler 480 Probes Master and Universal ProbeLibrary Probes (Roche, Mannheim, Germany). The conditions for PCR were as follows: 95°C for 10 min (1-cycle), 95°C for 10s, 60°C for 30s (single; 45-cycle quantification), 40°C for 10s (1-cycle). Sample quantifications were normalized to glyceraldehyde-3-phosphate dehydrogenase (GAPDH) expression. Primers were obtained from TIB Molbiol (Berlin, Germany). (GAPDH: sequence F: 5′- TGGGAAGCTGGTCATCAAC -3′; R: 5′- GCATCACCCCATTTGATGTT -3′; UPL probes 111 and caspase-12: sequence F: 5′- TGGATACTCAGTGGTGATAAAGGA -3′; R: 5′- ACGGCCAGCAAACTTCATTA -3′; UPL probes 94). The evaluation was performed with LightCycler 480 SW 1.5 software (Roche, Mannheim, Germany).

Caspase-12 immunolabeling

Immunoreactivity to caspase-12 (1:100; Novus Biologicals, Littleton, CO) was tested on buffered paraformaldehyde solution (4%) fixed, paraffin embedded 5-µm thick aortic sections. A semi-quantification assessment was performed on the basis of intensity and the distribution of labelling of the target protein, under a microscopic examination field using a digital camera. The intensity score values were as follows: 0: no positive staining, 1: weak staining, 2: moderate staining, 3: strong staining and the area score as: 1: up to 10% positive cells, 2: 11-50% positive cells, 3: 51-80% positive cells, 4: >80% positive cells. An average score was calculated for the whole picture (intensity score multiplied by area score, 0-12). Finally, each specimen was characterized with the average score of the four adjacent fields in a blinded fashion.

REFERENCES

1. Korkmaz-Icoz S, Brlecic P, Ruppert M, et al. Mechanical pressure unloading therapy reverses thoracic aortic structural and functional changes in a hypertensive rat model. *J Hypertens.* 2018.
